# The Clinical Application of Hormones

**Published:** 1914-03-14

**Authors:** 


### MODERN METHODS.

### The Clinical Application of Hormones.

Although so far but little scope has been found for the administration of hormones, there is no doubt that the present attention that is being directed to the investigation of the problems surrounding their formation and physiological function will be productive. Practitioners who are prevented from keeping in touch with modern physiological research may be reminded that the hormones are substances secreted by various glands, which have the power of exciting further activity in organs remote from the seat of their secretion. Thus secretin—formed by appropriate stimulation of the mucous membrane of the duodenum and jejunum—when absorbed into the blood-stream and conveyed thereby to the pancreas, quickly excites secretion by that organ. That is to say, the presence of food in the upper part of the small intestine sets in motion a mechanism by which the digestive juice required to render it capable of absorption is readily poured out. In other words, soon after the contents of the stomach are passed on to the duodenum these "chemical messengers"—hormones—are despatched from the latter to stir up the waiting pancreas. In this country Bayliss and Starling have made a special study of the hormones, the experiments carried out by them some twelve years ago which resulted in the discovery of the aforementioned pancreatic excitant, secretin, being classical. It is clear, then, that in such mechanisms we have means for co-ordinating the mutual relationships between various organs of the body, apart from the usual nervous reflexes; and whilst the circulation of the blood suggests a means by which the organic functions might thus be correlated apart from the workings of the nervous system, it is obvious that the ductless glands with their internal secretions may well supply some of the balancing agents.

In clinical medicine careful comparison of physical signs with structural changes and *post-mortem* findings continually discloses fresh relationships between organs; thus the form of pituitary disease described by Frölich in 1901 under the name of dystrophia adiposogenitalis revealed new facts as to the part of the pituitary gland in metabolism.

### Therapeutic Considerations.

The hormones which have been most closely studied include adrenalin, thyroid extract, secretin, and pituitrin, all of which have been shown to play a most important part as "chemical messengers" which stimulate various organic activities, and hence have aptly been given their present name, derived from a Greek word meaning "to excite."

Whilst the main clinical application of our increasing knowledge of hormones still remains, the administration of thyroid extract to patients suffering from deficient thyroid or pituitary secretion, and of adrenalin to cause vaso-constriction, the hormone of the pituitary body—pituitrin—is now coming into more frequent use. Pituitrin has been found successful in combating shock and various conditions of lowered blood pressure. Another hormone coming into use is chiefly known by a proprietary name, and has the power of increasing peristalsis of the intestine. A London physician, who is at present investigating the properties of this particular preparation, but who does not yet wish to give a definite statement as to his findings, is nevertheless able to report that injection of this hormone has at his hands certainly relieved several cases of chronic constipation which had resisted all other methods of treatment.

What is now wanted to increase our knowledge of the clinical uses of hormones are reports from men in general practice who have made a point of administering these substances in large series of suitable cases and made careful records of results.

### Progress of Research.

In view of the present additions to our knowledge of the hormones, it is interesting to recall that it is nearly seventy years since the true nature of the latter was first revealed by the experiments of Professor Berthold of Göttingen, whilst it is nearly thirty years since Marie described the now well-known condition of acromegaly, subsequently proved to be associated with pathological changes in the pituitary body. Now investigations are showing that hormones are formed by many, if not all, the glandular organs, and have led to the anticipation that organic extracts may be obtained which will exert a curative effect in various constitutional diseases, such as Bright's disease and chlorosis. Further clinical applications of hormone therapy may be suggested by Starling's discovery, confirmed by Biedle, of the hormonic relations between the tissues of the fœtus and the mammary changes of pregnancy.

An interesting field of research which is now being extensively worked concerns the pituitary body, about which our knowledge of pituitrin, referred to above, probably represents but a small part of its influence on growth and metabolism. Both histologically and embryologically the anterior and posterior portions of the pituitary body are dissimilar, yet although this organ is known to be concerned in the production of both acromegaly and gigantism, experimental injections of extracts from either part have failed to reveal just what that influence is. These abnormal states are since considered to be due to excessive action of the pituitary body—that is, to over-production of its hormones—a state of things which clinics are now familiar with under the term of hyper-pituitrism. On the other hand, the syndrome of Frölich, associated with pituitary tumour—infantile mentality, immature sexuality, and feminine build of body—is considered to be the result of hypo-pituitrism. Moreover, confirmation of the theory that not only growth and development, but fat and sugar metabolism, as well as the tone of involuntary muscles, are under pituitary control, will open the way to many further uses of pituitary hormones.

